# Malignant Small-Pox, Treated with Sulphur Fumigations, Etc.

**Published:** 1871-12

**Authors:** F. Hjaltelin

**Affiliations:** Chief Physician of Iceland, Rekjavik


### MALIGNANT SMALLPOX TREATED WITH SULPHUR FUMIGATIONS, AND SULPHUROUS ACID INTERNALLY.

By F. HJALTELIN, M. D., Chief Physician of Iceland, Rekjavik.

EVERY summer, and even early in the spring, Iceland is generally surrounded by many hundred French fishing-vessels, which, to tell the truth, are never welcomed by the Icelanders; this spring, these vessels were still more dreaded than ever, because accounts had been received of a malignant smallpox, raging over the unhappy French country.

In the beginning of April last, several French vessels came into the harbor of Rekjavik, and in some of these a third part of the crew had been seized by smallpox, and some had died. A quarantine was at once erected in the neighborhood of our town, and, as the chief physician of this island, I had to treat the sick sailors, and see that the quarantine regulations were strictly followed. Many of the patients brought into the quarantine had the confluent smallpox, and were in great danger. I treated them all in the same manner, viz., on the plan of disinfection advocated by me a long time ago. Sulphur fumigations were used in the sick room and a mixture of sulphurous acid in water was given internally. The result has been most satisfactory, and I shall give a full account of the cases treated, in Dr. Dobell's Reports this year. As no cases of smallpox have, after the lapse of thirty days, occurred among the Icelandic populution, either in the town or its neighborhood, I entertain the hope that the disease may be looked upon as stamped out by the aforesaid precautions. The people have been vaccinated and re-vaccinated, as a matter of course, and this may serve to protect the Icelandic population from this dreadful malady.—*Boston Medical and Surgical Journal.*

